# 1-Deoxynojirimycin Alleviates Insulin Resistance via Activation of Insulin Signaling PI3K/AKT Pathway in Skeletal Muscle of *db/db* Mice

**DOI:** 10.3390/molecules201219794

**Published:** 2015-12-04

**Authors:** Qingpu Liu, Xuan Li, Cunyu Li, Yunfeng Zheng, Guoping Peng

**Affiliations:** 1 College of Pharmacy, Nanjing University of Chinese Medicine, Nanjing 210023, China; lqpcy1224@163.com (Q.L.); xuanli@njutcm.edu.cn (X.L.); licunyuok@163.com (C.L.); zyunfeng88@126.com (Y.Z.); 2 Jiangsu Collaborative Innovation Center of Chinese Medicinal Resources Industrialization, Nanjing 210023, China

**Keywords:** mulberry leaves, 1-deoxynojirimycin, *db/db* mice, insulin resistance, insulin signaling pathway, skeletal muscle, PI3K/AKT, GLUT4 translocation

## Abstract

1-Deoxynojirimycin (DNJ) is widely used for the treatment of diabetes mellitus as an inhibitor of intestinal α-glucosidase. However, there are few reports about its effect on insulin sensitivity improvement. The aim of the present study was to investigate whether DNJ decreased hyperglycemia by improving insulin sensitivity. An economical method was established to prepare large amounts of DNJ. Then, *db/db* mice were treated with DNJ intravenously (20, 40 and 80 mg·kg^−1^·day^−1^) for four weeks. Blood glucose and biochemical analyses were conducted to evaluate the therapeutic effects on hyperglycemia and the related molecular mechanisms in skeletal muscle were explored. DNJ significantly reduced body weight, blood glucose and serum insulin levels. DNJ treatment also improved glucose tolerance and insulin tolerance. Moreover, although expressions of total protein kinase B (AKT), phosphatidylinositol 3 kinase (PI3K), insulin receptor beta (IR-β), insulin receptor substrate-1 (IRS1) and glucose transporter 4 (GLUT4) in skeletal muscle were not affected, GLUT4 translocation and phosphorylation of Ser473-AKT, p85-PI3K, Tyr1361-IR-β and Tyr612-IRS1 were significantly increased by DNJ treatment. These results indicate that DNJ significantly improved insulin sensitivity via activating insulin signaling PI3K/AKT pathway in skeletal muscle of *db/db* mice.

## 1. Introduction

Type 2 diabetes mellitus, the fourth leading cause of death worldwide, is a chronic metabolic disorder characterized by impaired homeostasis of lipid and carbohydrate metabolism, and ultimately results in insulin resistance and subsequent hyperglycemia [1]. The disease leads to several long-term complications such as retinopathy, nephropathy, neuropathy, hypertension, atherosclerosis and hyperlipidemia [2,3,4]. It affected an estimated366 million people in 2011 and this is expected to increase to 600 million in 2035 [5,6,7]. Type 2 diabetes mellitus is characterized by the loss of sensitivity to insulin, thus improving insulin resistance is an effective strategy to treat type 2 diabetes mellitus [8]. Chinese medicine is a rich source of natural drugs, and different parts of medicinal plants and their isolated materials have been found to be effective against diabetes.

Mulberry is a well-known deciduous tree, belonging to the genus *Morus* of the *Moraceae* family. Mulberry leaves are widely used in traditional Chinese medicine as functional or medical additive to control blood glucose [9,10,11,12]. The active constituents of mulberry leaves are flavonoids, alkaloids, steroids, and coumarins. Among these constituents, the antihyperglycemic effect is attributed mainly to alkaloids whose main compound is 1-Deoxynojirimycin (DNJ).

**Figure 1:**
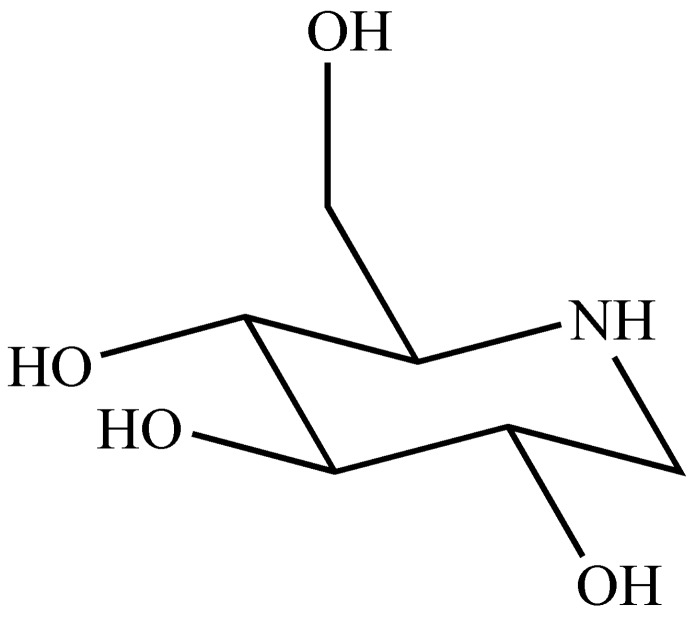
The chemical structure of 1-deoxynojirimycin.

DNJ is a glucose analogue with an NH group substituting for the oxygen atom of the pyranose ring (Figure 1). DNJ was first isolated from the mulberry leaves (*Morus alba* L.) by Yagi and coworkers [13]. DNJ is generally regarded as a competitive inhibitor of small intestinal brush-boarder α-glucosidase [14,15,16]. Therefore, the therapeutic consideration of DNJ always focuses on its postprandial hypoglycemic effect in the gastrointestinal tract. The bioactivity of DNJ in the digestive tract (α-glucosidase inhibition) has been investigated thoroughly, however, other effects of DNJ have been neglected. Kim *et al.* reported that DNJ could be absorbed into plasma in the intact form, and reached a maximum concentration 30 min after oral intake [17]. This prompted us to investigate whether DNJ has antidiabetic effect by improving insulin sensitivity.

Skeletal muscle is the primary and largest site of glucose disposal in the insulin-stimulated state [18,19,20,21,22]. Normally, insulin could lower blood glucose levels by facilitating glucose uptake into skeletal muscle. However, in an insulin resistant state, insulin-stimulated glucose disposal in skeletal muscle is severely damaged, as this organ does not respond to insulin properly, leading to a defect in the insulin-signaling pathway in muscle and elevating blood glucose [23,24]. Hence, we chose skeletal muscle to investigate the mechanism of DNJ alleviating insulin resistance in our study.

Therefore, the current study aimed to investigate whether DNJ could improve insulin sensitivity. To achieve this, DNJ was given to *db/db* mice, a reliable type 2 diabetic animal model, intravenously to investigate its effects and the related mechanisms in skeletal muscle.

## 2. Results and Discussion

In the present study, we established an economical method to prepare large quantities of DNJ. We then investigated its antidiabetic effects on *db/db* mice. We found that DNJ could improve type 2 diabetes by ameliorating insulin resistance. Furthermore, we explored the related molecular mechanisms in skeletal muscle.

### 2.1. Preparation of DNJ

The antihyperglycemic effect of mulberry leaves is attributed mainly to DNJ, however the amount of DNJ in mulberry leaves is very low, the content is only 0.1%, and it is difficult to prepare [12,25]. Hence, the compound is very expensive and this limits its research *in vivo*. In this study, we established an economical method to prepare large amounts of DNJ.

**Figure 2:**
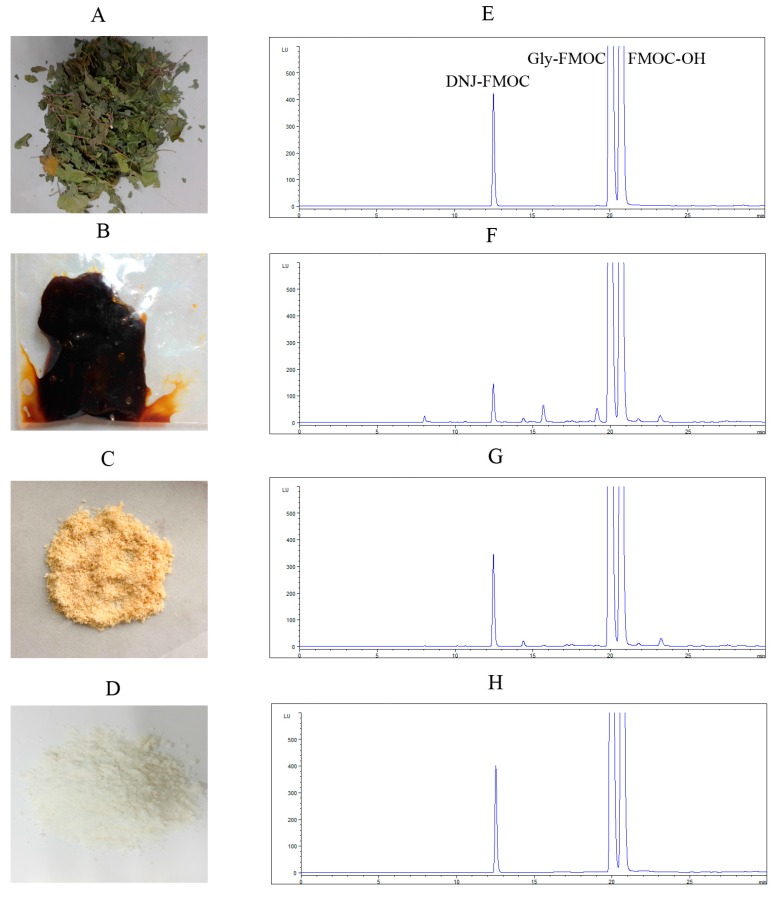
Preparation of DNJ: Dried mulberry leaves (**A**) was extracted by boiled water, purified by citation exchange resin and anion exchange resin (**B**); purified by silica gel H (**C**); and crystallized in 95% ethanol (**D**) successively; (**E**–**H**) Chromatograms of DNJ standard (**E**); sample purified by ion exchange resin (**F**); silica gel H (**G**); and the final product (**H**). DNJ was made to react with fluorenylmethoxycarbonyl chloride (FMOC-Cl) to generate the DNJ–FMOC derivative and detected by High Performance Liquid Chromatography coupled with fluorescence detector (HPLC-FLD).

As shown in Figure 2, DNJ was first extracted from dried mulberry leaves with boiling water; the extract was purified by cation exchange resin, anion exchange resin and silica gel H successively. Finally, DNJ was obtained after crystallized in 95% ethanol. We determined DNJ by High Performance Liquid Chromatography (HPLC) coupled with fluorescence detector. The product purified by silica gel H looked purer than that only purified by exchange resin. After these steps, the recovery of DNJ was over 50%. Then, the product was crystallized in 95% ethanol, and the purity of DNJ was over 95% in the final product. Although the recovery by crystallization process was only 30%, this method was very economical and the purity was satisfactory. Cation exchange resin was always used in purified alkaloids. Wang *et al.* used 732 resins to separate DNJ from other components in mulberry leaves extracts, the purity of DNJ in the final product was 15.3% [26]. In this study, we further used silica gel H to purify DNJ and crystallized in 95% ethanol successfully. The method can prepare large amounts of DNJ economically.

### 2.2. Effectsof DNJ on Body Weight and Average Food Intake, Water Intake, and Urine Output

**Figure 3:**
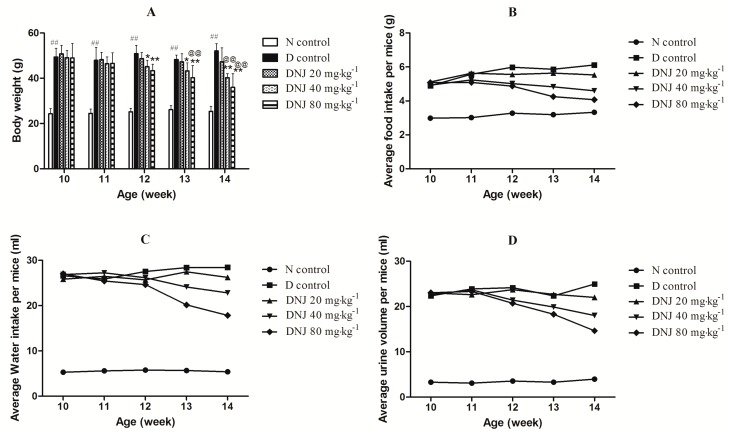
Effects of DNJ on body weight (**A**); and average food intake (**B**); water intake (**C**); and urine output (**D**) of *db/db* mice. Normal saline or DNJ were administered intravenously to N control mice or *db/db* mice for four weeks. ^##^ *p* < 0.01 *vs.* N control; * *p* < 0.05, ** *p* < 0.01 *vs.* D control; ^@@^ *p* < 0.01 *vs.* DNJ 20 mg·kg^−1^·day^−1^ (*n* = 6).

Body weight and average food intake, water intake, and urine output were monitored once a week during the experimental period. Body weight and average food intake, water intake, urine output of D control (diabetic control) were significantly higher than that of N control (normal control) mice. As shown in Figure 3A, although there was no significant difference in the first week (11th week) of treatment, the body weight of *db/db* mice treated with DNJ decreased significantly in a dose-dependent manner compared with D control from the second week (12th week) of treatment. Average food intake, water intake, and urine output of *db/db* mice treated with DNJ were also reduced compared with D control group (Figure 3B–D).

Regarding the role of DNJ in weight control, Kong *et al.* have reported the anti-obesity effect of DNJ in Otsuka Long-Evans Tokushima Fatty (OLETF) rats [27]*. Bacillus subtilis* MORI-fermented soybean extract, which contains DNJ, decreased body weight due to its effects on lipid metabolism in *db/db* mice [28]. In our study, DNJ treatment significantly decreased body weight of *db/db* mice in a dose-dependent manner. *Db/db* mice are type 2 diabetic animal model associated with obesity, beginning at an early age, *db/db* mice are extremely obese [29]. Notably, the presence of these conditions synergistically increases the risk of type 2 diabetes [30]. Obese and overweight are more likely to develop insulin resistance; therefore, losing weight may help the treatment of type 2 diabetes.

### 2.3. DNJ Protecteddb/db Mice against the Onset of Type 2 Diabetes

**Figure 4:**
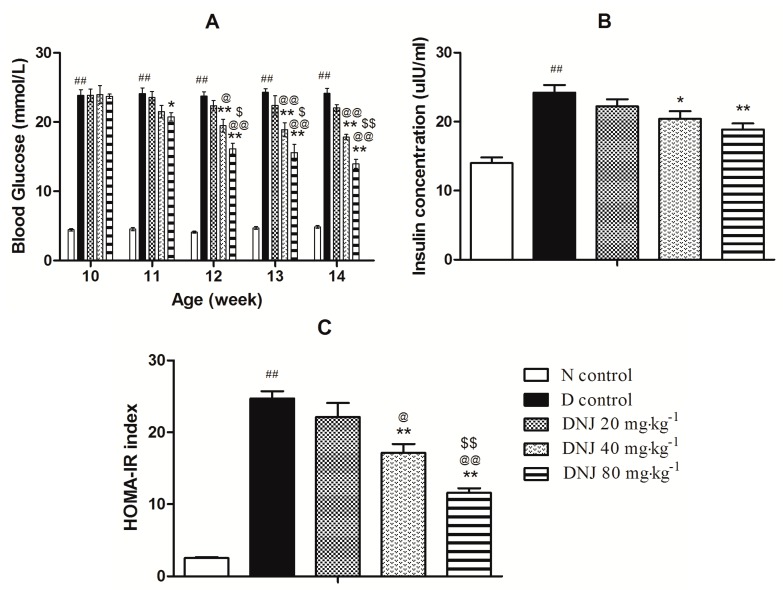
Antidiabetic effect of DNJ on *db/db* mice. Blood glucose (**A**) and serum insulin (**B**) were determined. HOMA-IR index (**C**) was calculated. ^##^ *p* < 0.01 *vs.* N control; * *p* < 0.05, ** *p* < 0.01 *vs.* D control; ^@@^ *p* < 0.01 *vs.* DNJ 20 mg·kg^−1^·day^−1^; ^$^ *p* < 0.05, ^$$^ *p* < 0.01 *vs.* DNJ 40 mg·kg^−1^·day^−1^ (*n* = 6).

As shown in Figure 4A, *db/db* mice developed a stable high blood glucose, DNJ treatment significantly reduced blood glucose levels in a dose-dependent manner from the 11th week compared with D control group. Furthermore, serum insulin level of *db/db* mice was higher than that of N control mice. Insulin plays a critical role in the maintenance of blood glucose homeostasis. However, in an insulin resistant state, insulin does not promote glucose uptake and utilization effectively, so insulin secretion increases excessively with compensation to maintain blood glucose homeostasis [31]. However, DNJ (40 and 80 mg·kg^−1^·day^−1^) treatment significantly reduced serum insulin levels (Figure 4B). Moreover, the homeostasis model assessment insulin resistance (HOMA-IR) index, a parameter for evaluating the degree of insulin resistance, were markedly enhanced in D control mice (Figure 4C), and it was significantly reversed in a dose-dependent manner by the administration of DNJ for four weeks.

DNJ was reported to improve diabetic conditions by inhibiting the activity of α-glucosidase and the absorption of glucose in the intestinal brush border [32,33]. DNJ was demonstrated to improve the glycemic control in alloxan-induced diabetic mice and in STZ-induced diabetic rats [34,35]. Kong *et al.* reported that DNJ could improve insulin resistance and/or insulin sensitivity in OLETF rats [27]. Here, we firstly demonstrated that DNJ ameliorated insulin resistance in *db/db* mice. In our study, DNJ treatment significantly reduced blood glucose, serum insulin level and HOMA-IR index. These results indicated that DNJ had a significant therapeutic action against type 2 diabetes by ameliorating insulin resistance.

### 2.4. Effect of DNJ on IPGTT and IPITT

**Figure 5:**
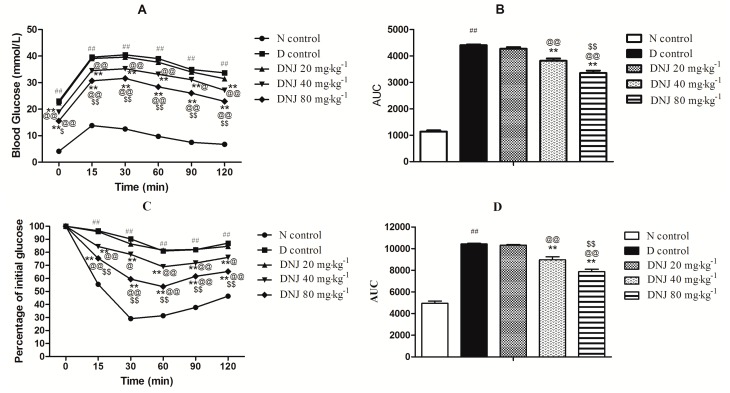
Intraperitoneal Glucose Tolerance Test (IPGTT) (**A**) and Intraperitoneal Insulin Tolerance Test (IPITT) (**C**) were determined, and Area under the curve (AUC) levels of IPGTT (**B**) and IPITT (**D**) were calculated. ^##^ *p* < 0.01 *vs.* N control; ** *p* < 0.01 *vs.* D control; ^@^ *p* < 0.05, ^@@^ *p* < 0.01 *vs.* DNJ 20 mg·kg^−1^·day^−1^; ^$^ *p* < 0.05, ^$$^ *p* < 0.01 *vs.* DNJ 40 mg·kg^−1^·day^−1^ (*n* = 6).

Glucose tolerance and insulin tolerance tests were carried out in consideration of type 2 diabetes features, such as glucose intolerance and insulin resistance [31]. As shown in Figure 5A,B, the glucose tolerance was described by an intraperitoneal glucose tolerance test, and a damaged glucose tolerance in *db/db* mice was found, which was significantly improved in a dose-dependent manner by DNJ treatment. Area under the curve (AUC) of DNJ treatment was significantly decreased in a dose-dependent manner compared with D control. For IPITT, there was also an impaired insulin intolerance observed in the *db/db* mice and reversed by DNJ treatment (Figure 5C,D) in a dose-dependent manner.

It was reported that DNJ treatment showed significant improvements in glucose tolerance of OLETF rats [27]. Li *et al.* reported that DNJ could improve the glucose tolerance of alloxan-induced diabetic mice [34]. In this study, the results of IPGTT and IPITT showed significant improvements in glucose tolerance and insulin tolerance in response to DNJ. Combined with the reduction in body weight, blood glucose levels and serum insulin levels, these results clearly indicated an improvement in insulin resistance and/or insulin sensitivity in *db/db* mice treated with DNJ.

Higher doses of DNJ (40 and 80 mg·kg^−1^·day^−1^) have significant therapeutic effects. It would require hundreds of grams of Mulberry leaves per day to reach these concentrations, as the DNJ content is too low. However, these concentrations could be reached easily through diet of DNJ or the alkaloids extracted from Mulberry leaves.

### 2.5. Effect of DNJ onGLUT4 Protein Expression and Translocation

Glucose transporters play an important role in the regulation of blood glucose levels. Glucose transporter 4 (GLUT4) was specifically expressed in skeletal muscle and adipose tissue, where it took up glucose to reduce hyperglycemia [36].

**Figure 6:**
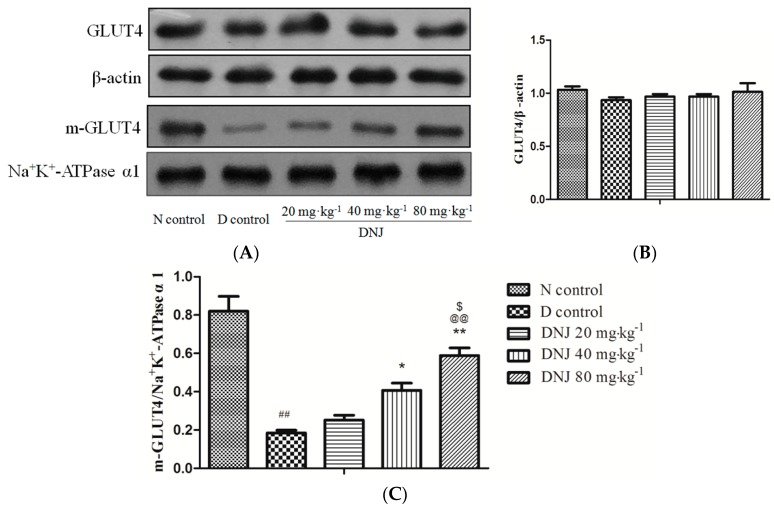
Effects of DNJ on GLUT4 expression and translocation in *db/db* mice. Western blot analysis for GLUT4 and m-GLUT4 protein expression in skeletal muscle of *db/db* mice (**A**). Expression ratio of GLUT4 (**B**) and m-GLUT4 (**C**) were analyzed. These data were representative of three independent experiments. The GLUT4 protein was normalized by β-actin and the m-GLUT4 was normalized by Na^+^K^+^-ATPase α1. ^##^ *p* < 0.01 *vs.* N control; * *p* < 0.05, ** *p* < 0.01 *vs.* D control; ^@@^ *p* < 0.01 *vs.* DNJ 20 mg·kg^−1^·day^−1^; ^$^ *p* < 0.05 *vs.* DNJ 40 mg·kg^−1^·day^−1^ (*n* = 3).

To understand whether DNJ reduced blood glucose level via promoting total GLUT4 protein expression or increasing translocation of GLUT4 (m-GLUT4), we analyzed total GLUT4 and m-GLUT4 expression levels in skeletal muscle by Western blot. As shown in Figure 6, there were no significant differences in total GLUT4 protein between N control mice and D control mice, but the m-GLUT4 protein in *db/db* mice was dramatically reduced; these results were consistent with the study that reported that the total GLUT4 protein expression was unchanged between insulin resistant and control mice [37]. However, DNJ treated *db/db* mice showed higher (almost three times) expression of m-GLUT4 than D control (*p* < 0.01).

Lee *et al.* proved that decreased GLUT4 translocation in epididymal skeletal muscle elevated blood glucose level of obese diabetic *ob/ob* mice [38]. It was reported that increased GLUT4 translocation improved hyperglycemia in type 2 diabetic GK rats and KKAy mice [39]. In this study, our results indicated that DNJ alleviated hyperglycemia via promoting GLUT4 translocation to the plasma membrane rather than via up-regulating GLUT4 concentration.

### 2.6. DNJ Up-Regulated Phosphorylation of AKT, PI3K, IR-β, and IRS1

Insulin promotes glucose uptake via activating a series of signaling cascades initiated by insulin binding. In this pathway, binding of insulin to the insulin receptor (IR) activates phosphorylation of the tyrosine kinase domain, followed by tyrosine phosphorylation of insulin receptor substrates (IRS). Phosphorylation of IRS interacts with the p85 regulatory subunit of phosphoinositide3-kinase (PI3K), leading to the activation of the enzyme and phosphorylation Ser473-AKT, which then phosphorylates, and subsequently actives stimulation of GLUT4 vesicle translocation to the plasma membrane and ultimately induces enhancement of glucose transport [40,41,42,43,44,45,46]. Therefore, as insulin-stimulated GLUT4 translocation was increased by DNJ treatment in *db/db* mice, we subsequently evaluated the expressions of total AKT, PI3K, IR-β and IRS1 as well as their phosphorylation in skeletal muscle of *db/db* mice, which are the major upstream regulators of GLUT4 translocation.

**Figure 7:**
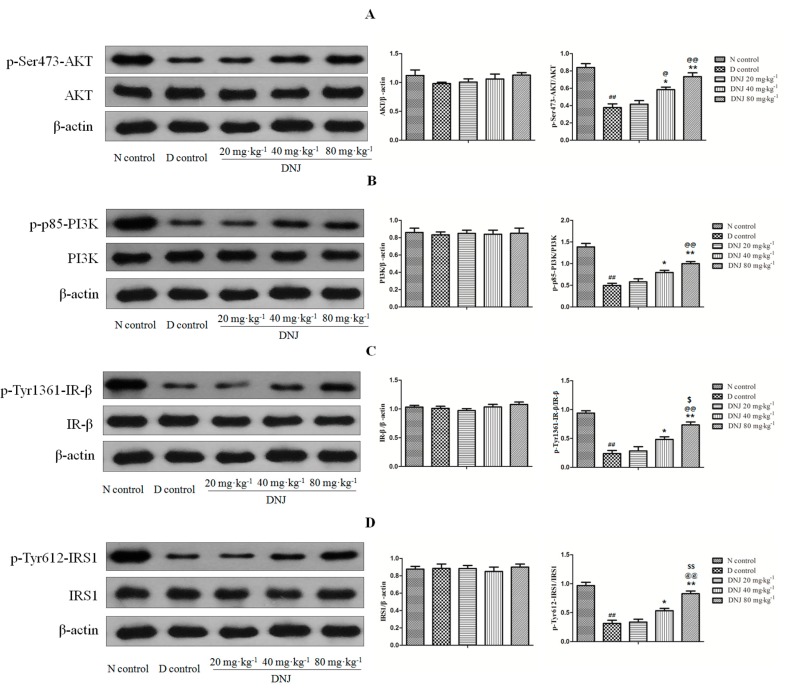
Effects of DNJ on expression of AKT, PI3K, IR-β, and IRS1 and their phosphorylation in *db/db* mice. Western blot analysis for p-AKT and AKT (**A**); p-PI3K and PI3K (**B**); p-IR-β and IR-β (**C**); and p-IRS1 and IRS1 (**D**) in skeletal muscle of *db/db* mice. These data were representative of three independent experiments. Results were normalized by β-actin, ^##^ *p* < 0.01 *vs.* N control; * *p* < 0.05, ** *p* < 0.01 *vs.* D control; ^@^ *p* < 0.05, ^@@^ *p* < 0.01 *vs.* DNJ 20 mg·kg^−1^·day^−1^; ^$^ *p* < 0.05, ^$$^ *p* < 0.01 *vs.* DNJ 40 mg·kg^−1^·day^−1^ (*n* = 3).

As shown in Figure 7A,B, there were no significant differences in total AKT and PI3K protein contents between N control mice and D control mice. The phosphorylation of AKT and PI3K were decreased in skeletal muscle of D control mice. However, the phosphorylation of AKT and PI3Kwere greatly increased by DNJ treatment compared with the D control group. As DNJ up-regulated the phosphorylation of AKT and PI3K, we then investigated the total protein and tyrosine phosphorylation of IR-β and IRS1, which could activate PI3K and AKT. Total IR-β and IRS1 remained unchanged between N control mice and D control mice and the phosphorylation of IR-β, IRS1 were decreased in skeletal muscle of D control mice. However, treatment with DNJ remarkably enhanced the phosphorylation of IR-β and IRS1 (Figure 7C,D). These results clearly indicated that DNJ increased GLUT4 translocation to the plasma membrane via up-regulating the phosphorylation of IR-β, IRS1, AKT and PI3K rather than increasing the expression of total IR-β, IRS1, AKT and PI3K.

Blood glucose levels were decreased by insulin via facilitating glucose uptake mainly into fat tissue and skeletal muscle normally. However, in an insulin resistant state, skeletal muscle do not properly respond to insulin, consequently causing a reactive increase in insulin secretion by the pancreatic β-cells and hyperglycemia [47]. The phosphorylation of Tyr1361-IR-βand Tyr612-IRS1 were decreased in skeletal muscle of diet-induced hyperglycemic mice but their total protein were unchanged [48], and the PI3K/AKT pathway was not activated by insulin as much as under normal conditions [45]. These reports are consistent with that of the present study. Enhancement of AKT phosphorylation in skeletal muscle significantly improved hyperglycemia and insulin resistance of type 2 diabetic mice [49]. Consequently, the role of phosphorylation of IR-β, IRS1, PI3K and AKT in signaling pathways is very crucial in anti-hyperglycemia and insulin sensitivity. In this study, the expression levels of total IR-β, IRS1,PI3K, and AKT were not changed; however, insulin stimulated phosphorylation of Tyr1361-IR-β, Tyr612-IRS1, p85-PI3K, and Ser473-AKT were increased by DNJ treatment in the skeletal muscle of *db/db* mice. These results demonstrated that DNJ improved insulin sensitivity via activating phosphorylation of IR-β and IRS1, which subsequently increased phosphorylation of PI3K, AKT and finally enhanced GLUT4 translocation in skeletal muscle of *db/db* mice.

In summary, body weight, blood glucose, serum insulin levels and HOMA-IR index of the *db/db* mice treated with DNJ decreased significantly compared to *db/db* mice treated with normal saline. Furthermore, glucose tolerance and insulin sensitivity were improved in *db/db* mice treated with DNJ. Moreover, the reduction in GLUT4 translocation and the down-regulated phosphorylation of IR-β, IRS, PI3K and AKT in skeletal muscle of *db/db* mice were restored by DNJ treatment.

## 3. Experimental Section

### 3.1. Materials

Mulberry leaves were purchased from the city of Bozhou, Anhui Province, China. Ion exchange resins including 001 × 7 and 201 × 4 were purchased from Cangzhou Bon Adsorber Technology Co., Ltd. (Cangzhou, China). The DNJ standard was purchased from J & K Scientific Ltd. (Beijing, China). Glucose monitors were purchased from ACCU-CHEK^®^ (Shanghai, China). Insulin ELISA Assay Kit was purchased from Nanjing Jiancheng Bioengineering Institute (Nanjing, China). Bicinchonininc acid protein assay was purchased from Beyotime Institute of Biotechnology (Nanjing, China). IR-β, p-Tyr1361-IR-β, p-p85-PI3K, p-Tyr612-IRS1, and GLUT4antibodies were provided by ABCAM (Cambridge, UK). PI3K, AKT, p-Ser473-AKT and β-actin antibodies were purchased from Cell Signaling Technology (Danvers, MA, USA). IRS1, Na^+^K^+^-ATPase α1 was products of EnoGene (New York, NY, USA), HRP conjugated antibody IgG was an ABCAM product. ECL detection kit was provided by Thermo Electron Corp., (Rockford, IL, USA). Mammalian Membrane Protein Extraction Kit was purchased from Enjing (Nanjing, China).

### 3.2. Preparation of DNJ

Dried mulberry leaves were extracted in boiled water 1 h for two times, the extract water was adjusted to 3–4 by using 37% HCl (*v*/*v*), and passed over a cation exchange resin (001 × 7) column. The cation exchange resin was eluted with ethanol-water (70:30, *v*/*v*) and ammonia (4%, *v*/*v*), successively, the gathered ammonia effluent was then subjected to an anion exchange resin (201 × 4) column, eluented by water, and both the effluent and concentrate were stored under vacuum. Then, the product was subjected to a silica gel H column. This included dry packing—the sample was dissolved in a small amount of water and three times the sample of silica gel H was added to this solution. The solvent was then removed under vacuum in a rotary evaporator leaving the sample adsorbed on to the silica gel. This dry silica gel H containing the sample was transferred to the top of the column bed and eluted with 95% ethanol, and the eluent and concentrate were stored under vacuum, and finally crystallized in 95% ethanol.

### 3.3. Quantitative Determination of DNJ

DNJ was determined by High Performance Liquid Chromatography coupled with fluorescence detector (HPLC-FLD) [50]: Derivatization: Ten microliters of DNJ standard solution or sample was mixed with 10mL of 0.4 M potassium borate buffer (pH 8.5) in a 1.5 mL microtube. Twenty microliters of 5mM FMOC-Cl in CH-CN was added with immediate mixing and allowed to react at 20 °C for 20 min in a water circulator. Ten microliters of 0.1 M glycine was added to terminate the reaction by quenching the remaining FMOC-Cl. The mixture was diluted with 950 mL of 0.1% (*v*/*v*) aqueous acetic acid (17.5 mM) to stabilize the DNJ-FMOC, and filtered through a 0.2-mm nylon syringe filter. A 10-mL aliquot of the filtrate was injected into the HPLC system.

Chromatographic separation was performed on a Thermo Scientific C18 reversed-phase column (5 μm, 250 mm × 4.6 mm) for HPLC analysis. The mobile phase consisted of A (acetonitrile) and B (0.1% acetic acid/water) using a gradient elution 25%–65% A at 0–25 min and 65% A at 25–30 min. The flow rate was kept at 1.0 mL/min and column temperature was kept at 25 °C. The fluorescence detector was set at 254 nm for excitation wavelength and 322 nm for emission wavelength.

### 3.4. Animals

Male wild-type C57BLKS mice and C57BLKS/*Lepr*^db^ (*db/db*) mice were purchased from Model Animal Research Center of Nanjing University (MARC; Nanjing, China) at nine weeks, and all animals were maintained under a standard light (12 h light/dark) and temperature condition (22 ± 2 °C). The mice were fed a standard chow diet and mice had free access to food and water *ad libitum*. All experimental procedures were performed in accordance with the International Guidelines for Care and Use of Laboratory Animals and approved by the Animal Ethics Committee of Nanjing University of Chinese Medicine.

### 3.5. Experimental Design

**Figure A1:**
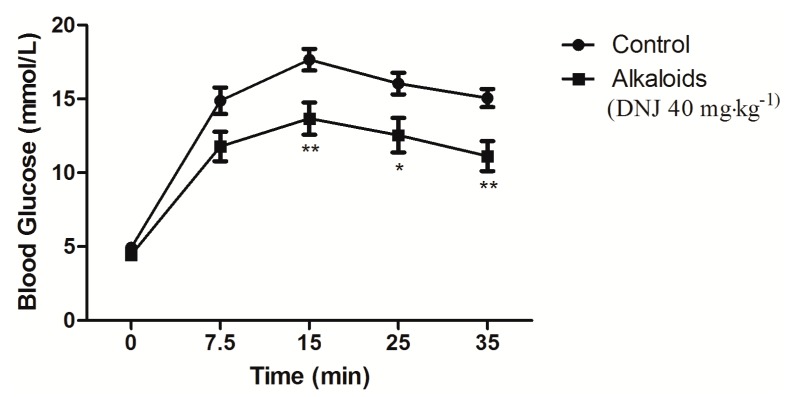
Effect of alkaloids (DNJ 40 mg·kg^−1^) isolated from Mulberry leaves on glucose tolerance test of ICR mice. * *p* < 0.05, ** *p* < 0.01 *vs.* control (*n* = 10).

**Figure A2:**
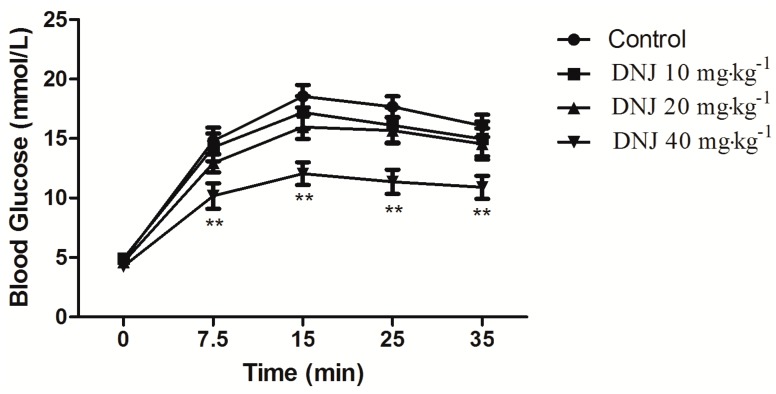
Effect of DNJ (10, 20, and 40 mg·kg^−1^) on glucose tolerance test of ICR mice. ** *p* < 0.01 *vs.* control (*n* = 10).

At the end of ten weeks, wild-type C57BLKS mice, which received intravenously normal saline, served as a normal control (N control) (*n* = 6). The *db/db* mice were divided into four groups (*n* = 6): Group I served as a diabetic control and received intravenously normal saline (D control). Group II, III, and IV were treated intravenously with DNJ 20, 40, and 80 mg·kg^−1^·day^−1^, respectively. An intravenous injection was selected to avoid the function of DNJ as an α-Glycosidase inhibitor inthe gastrointestinal tract. For DNJ doses selection, in our previous study, we screened a large number of Chinese traditional medicines including mulberry leaves by glucose tolerance test of ICR mice. We found the alkaloids (DNJ 40 mg·kg^−1^) isolated from mulberry leaves could improve the glucose tolerance test of ICR mice (Figure A1). We then tested doses of 10, 20, and 40 mg·kg^−1^, but both 10 and 20 mg·kg^−1^ did not have any effect (Figure A2). Therefore, we selected the DNJ doses as 20, 40, and 80 mg·kg^−1^·day^−1^. All these doses were given for 4 weeks. The blood glucose, body weight and average food intake, water intake, and urine output were measured every week. At the end of the experimental period, the mice were anesthetized with chloral hydrate after withholding food for 12 h, and blood samples were taken to determine the serum insulin levels. Besides, skeletal muscle were removed after the blood was collected, then rinsed with a physiological saline solution, and immediately stored at −80 °C.

### 3.6. Body Weight and Average Food Intake, Water Intake, Urine Output

The body weight of the mice was monitored weekly after removal of food for 2 h [51]. Average food intake, water intake and urine volume were measured weekly. Food intake was expressed as g·mice^−1^·day^−1^, water intake and urine output were expressed as mL·mice^−1^·day^−1^.

### 3.7. Glucose and Serum Insulin Measurements

Glucose measurement was performed on blood drawn from the tail vein using glucose monitors between 9:00 and 10:00 a.m. after a 12-h fasting period. The levels of plasma insulin were determined using an Insulin ELISA Assay Kit. Insulin resistance was assessed by a homeostasis model assessment of insulin resistance index (HOMA-IR) as previously described [52].

### 3.8. Intraperitoneal Glucose Tolerance Test (IPGTT) and Intraperitoneal Insulin Tolerance Test (IPITT)

IPGTT and IPITT were tested in the last week. For IPGTT, mice were fasted for 12-hour before mice were injected intraperitoneally with glucose at 0.5 g/kg body weight. For IPITT, mice were injected intraperitoneally with insulin at 0.5 U/kg body weight after a 4-h fast [51]. Glucose values were measured in whole venous blood using a blood glucose monitoring system at 0, 15, 30, 60, 90 and 120 min after the administration of either glucose or insulin.

### 3.9. Extraction of Membrane Protein from Skeletal Muscle

The extraction and isolation of membrane protein were performed according to the instructions for the *ProteinExt*™ Mammalian Membrane Protein Extraction Kit. Briefly, skeletal muscle was first lysed with Membrane Protein Extraction Buffer I supplemented with 1 mM PMSF. Then the samples were centrifuged for 15 min at 16,000× *g* at 4 °C. The supernatant was removed by centrifugation, and precipitate was lysed with Membrane Protein Extraction Buffer II supplemented with 1 mM PMSF. Finally, precipitate was lysed with Membrane Protein Extraction Buffer II supplemented with 1 mM PMSF and centrifuged for 15 min at 16,000× *g* at 4 °C. The precipitate is removed by centrifugation, and the supernatant was membrane protein.

### 3.10. Western Blot

In order to investigate the effects of DNJ on insulin signaling pathways, western blot analysis was performed as previously described [53]. Briefly, skeletal muscle tissues (0.1 g) were lysed in lysis buffer (50 mM Tris (pH 7.4), 150 mM NaCl, 0.1% SDS, 0.5% sodium deoxycholate, 1% NP40, 10 μL phosphatase inhibitors, 1 μL protease inhibitor and 5 μL 100 mM PMSF), centrifuged for 15 min at 16,000× *g* at 4 °C, and protein concentration was quantified by bicinchonininc acid protein assay. Equal amounts of protein (70 μg) were loaded on 10% SDS-PAGE and transferred onto PVDF membranes. After membranes were blocked, they incubated with the primary antibodies against IR-β, p-Tyr1361-IR-β, IRS1, p-Tyr612-IRS1, PI3K, p-p85-PI3K, AKT, p-Ser473-AKT, GLUT4, β-actin or Na^+^K^+^-ATPase α1 overnight at 4 °C followed by HRP conjugated secondary antibody for 2 h at room temperature. Protein bands were visualized using an ECL detection kit. Normalization of total protein expression was carried out by using β-actin as control. Normalization of m-GLUT4 expression was carried out using Na^+^K^+^-ATPase α1 as control [54].

### 3.11. Data Analysis

Data were expressed as mean ± SEM. One way analysis of variance (ANOVA), followed by Dunnett’s *post hoc* test was used to determine statistical differences. A *p*-value of less than 0.05 was considered statistically significant.

## 4. Conclusions

In this study, an economical method was established to prepare large amounts of DNJ, and the data presented in this paper demonstrated that DNJ alleviated hyperglycemia by improving insulin sensitivity via activating insulin signaling PI3K/AKT pathway in skeletal muscle of *db/db* mice. DNJ has been recognized as α-Glycosidase inhibitor for the past decades; however, our present study provided strong evidence that DNJ alleviates hyperglycemia by improving insulin sensitivity in skeletal muscle of *db/db* mice.
